# Effect of Laser Parameters on Optical Stealth Transmission System Performance

**DOI:** 10.3390/s21165358

**Published:** 2021-08-09

**Authors:** Xinmei Wang, Weifeng Mou, Huatao Zhu

**Affiliations:** College of Information and Communication, National University of Defense Technology, Changsha 410003, China; wangxinmei1990@163.com (X.W.); mouweifeng20@nudt.edu.cn (W.M.)

**Keywords:** semiconductor lasers, system performance, optical stealth transmission, gain-switching

## Abstract

The performance of an optical stealth transmission system based on gain-switched laser depends largely on the laser parameters. Modulation frequency, bias current, and modulation current are considered to study the covertness and bit error rate performance of the optical stealth transmission system. According to optical stealth carrier generation with time spreading and all-optical encoding, the stealth signals are derived. A complementary encoding scheme is adopted in the system simulation. The simulation results show that the temporal and spectral characteristics of the generated stealth signal can be changed by adjusting the bias current, modulation current, and modulation frequency. However, there is a trade-off between bit error rate performance and covertness of the stealth channel. Under the premise of error-free transmission, the bias current and modulation frequency should be reduced and the modulation current should be improved to optimize the covertness of the stealth channel.

## 1. Introduction

Laser source is one of the most important devices for optical communication systems, especially in optical secure communications, such as optical chaotic communications [1,2] and optical stealth communications [3,4]. Gain-switching has proven its convenience as a method for generating optical pulses due to its low cost, simplicity, and stability [5,6]. Especially, gain-switching with external injection is an attractive alternative for flexible optical frequency comb generation [7,8]. In optical secure communication, gain-switched optical pulses have been found to be applied in optical stealth transmission [9,10]. Signal characteristic in both the time and spectral domain depends on the laser parameter of the laser source, which affects the optical signal processing of the stealth signal in optical stealth communication.

In this paper, the influence of laser parameters on optical stealth transmission system performance is investigated for the first time. The optical stealth signal is encoded in the time and frequency domains. To avoid temporal energy fluctuation in the single-encoding scheme, a simple, complementary, encoded optical stealth transmission system is proposed and demonstrated. In the generation of an optical stealth signal, the laser parameters affect the bandwidth, tone-to-noise ratio, and pulse width of the generated optical pulses. Different to the work exploiting the optical frequency comb character of the gain-switched distributed feedback (DFB) laser, the gain-switched laser in the optical stealth transmitter works at a continuum spectra state with little tone-to-noise ratio (TNR). The bit error rate (BER) performance of the optical stealth channel is also influenced by the laser parameters of gain-switched laser in the optical stealth transmitter.

This paper is organized as follows. In the second section, the subsystem model of optical stealth transmitter is described, and the signals generated under different laser parameters are analyzed. A complementary encoded optical stealth transmission system is introduced in the third section. In the fourth section, the covertness and BER performance are studied by system simulations and the effect of laser parameters are evaluated and discussed. Conclusions are presented in the last section.

## 2. Generation of Optical Stealth Carrier

**Figure 1:**
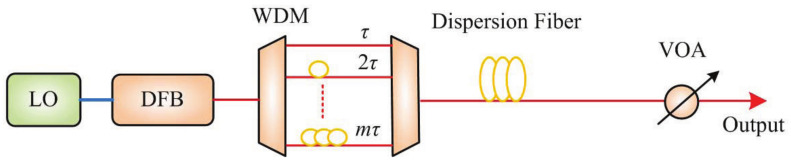
The generator of optical stealth carrier based on gain-switched optical pulses. LO, local oscillator; DFB, distributed feedback laser; WDM, wavelength division multiplexing; VOA, variable optical attenuator.

Based on gain-switched optical pulses, the configuration of optical stealth carrier generator is shown in Figure 1. The sine wave from local oscillator (LO) is launched into the DFB semiconductor laser. The gain-switched optical pulses are divided into different ports for encoding in the spectral domain. Each port is connected with an optical delay line to achieve time-domain encoding. The multiplexed signals are sent to a dispersion fiber span for temporal spreading. The transmitting power of stealth signal is controlled by a variable optical attenuator.

The interaction between photons and carriers in the DFB laser can be modeled by the rate equations [11,12,13]. The dynamics of carrier density N(t) and complex electric field E(t) in the DFB laser can be expressed as

(1) $$\begin{array}{c} \frac{dE(t)}{dt}=\frac{v_{g}a\left(N\left(t\right)-N_{t}\right)\Gamma E\left(t\right)}{2[1+\varepsilon E^{2}\left(t\right)]}-\frac{E(t)}{2\tau_{p}}+\frac{\Gamma \beta BN^{2}\left(t\right)}{2E(t)}+F_{E}\\ \frac{dN(t)}{dt}=\frac{I(t)}{eV}-\frac{v_{g}a\left(N\left(t\right)-N_{0}\right)}{1+\varepsilon E^{2}\left(t\right)}E^{2}\left(t\right)-\frac{N(t)}{\tau_{n}}+F_{N} \end{array}$$

where all the parameters have a unique meaning and some of them are listed in Table 1. In addition, I(t) is a time-varying injection current that contains the bias current $I_{b}$ and sinusoidally varying current $I_{m}$ from the LO. The Langevin noise sources $F_{E}$ and $F_{N}$ take into account spontaneous emission and spontaneous carrier recombination.

The power of the gain-switched optical pulses is given by

(2) $$P\left(t\right)=\frac{E^{2}\left(t\right)V\eta_{0}hv}{2\Gamma \tau_{p}}$$

where $\eta_{0}$ is the differential quantum efficiency, *h* is Planck’s constant, and *v* is the unmodulated optical frequency. Therefore, the spectrum of the optical pulses can be expressed as

(3) $$\Omega =\Omega_{0}+FFTshift\left(FFT\left|\sqrt{P}\exp(j\phi \left(t\right)\right|\right))$$

where $\Omega_{0}$ is the center angular frequency, FFT is fast Fourier transform, FFTshift shifts the outputs of FFT to the center of the spectrum, ϕ is the instantaneous phase term. Further, the complex electric field of the gain-switched optical pulses based stealth carrier can be described as

(4) $$e_{d}\left(t\right)=e\left(t\right)\exp\left(-j\psi \left(\Omega \right)\right)$$

where ψ is the phase delay induced by the chromatic dispersion of the dispersion fiber and e(t) can be written as

(5) $$e\left(t\right)=\sum_{i=1}^{m}\frac{1}{m}\sqrt{P}\exp\left(j\Omega_{i}\left(t-i\tau \right)+j\phi \right)$$

where *m* is the number of ports, $\Omega_{i}$ is the center angular frequency of *i*-th port, and τ is the time-slot duration.

**Figure 2:**
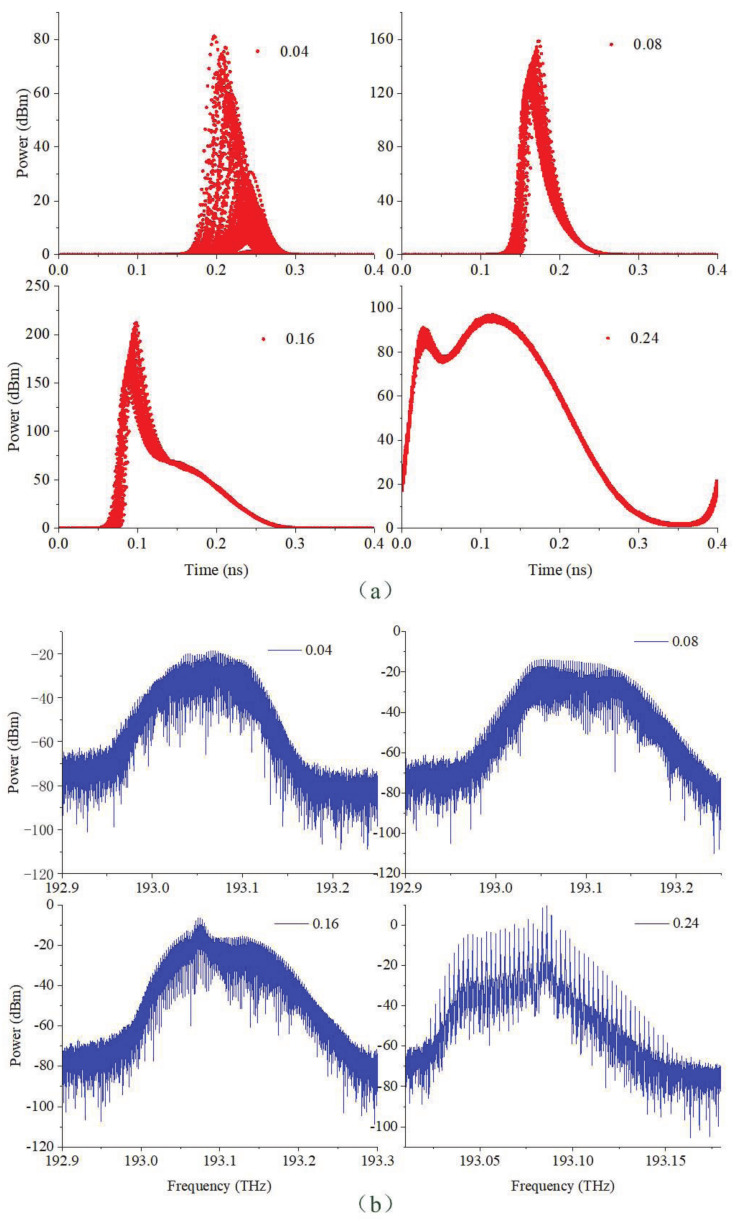
(**a**) The eye diagram and (**b**) the optical spectrum of the gain-switched optical pulse with different bias currents.

The gain-switched optical pulses with the different bias currents are shown in Figure 2. The modulation frequency is 2.5 GHz and the modulation current is 0.2 A. In Figure 2a, the bias current varies from 0.04 to 0.24 A. At 0.04 A, the relaxation oscillation frequency is 2.49 GHz, the generated optical pulses vary greatly in pulse position and amplitude. The modulation frequency is larger than the relaxation oscillation frequency, and the modulation current to bias current ratio is 5; the spike of relaxation oscillator is not entirely caught. Therefore, the eye diagram exhibits large time and amplitude jitters. The bias current is then switched to 0.08 A, and the relaxation oscillation frequency is 5.24 GHz. Gain-switched optical pulse with less jitters than that at 0.04 A is generated. As the bias current is increased to 0.16 A, the generated optical pulses display a tailing in the trailing edge. The relaxation oscillation frequency is 7.36 GHz and the second spike is excited with power lower than the first spike. Finally, with the bias current set to 0.24 A, the relaxation oscillation frequency is 7.98 GHz. Then, the power of the second spike is enhanced and the generated optical pulse exhibits a two-pronged shape.

The spectral output from the gain-switched laser is shown in Figure 2b. At 0.04 A, the optical spectrum shows obvious broadening due to frequency chirp. The frequency chirp across optical pulses is caused by a time-varying carrier density in the laser active region during the direct modulation. Then, at 0.08 A, the optical spectrum is broadened. At 0.16 A, a peak begins to appear in the spectrum due to the tailing in the optical pulses. Finally, discernible spectral lines appear in the optical spectrum, and the offset of each tone is an integral multiple of the modulation frequency.

**Figure 3:**
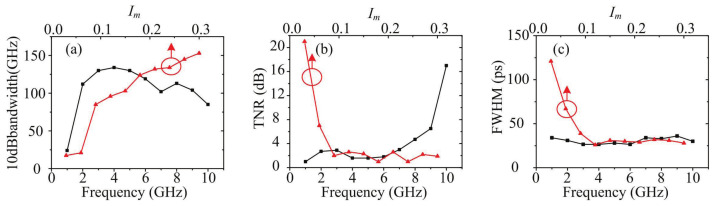
The (**a**) bandwidth, (**b**) TNR, (**c**) FWHM changes along with the frequency and modulation current.

The bandwidth, TNR, and full width at half maximum (FWHM) of the generated optical pulse with different modulation currents and frequencies are shown in Figure 3. The bias current is set to 0.08 A. In Figure 3a, the 10 dB bandwidth is proportional to the modulation current. During the tuning of modulation frequency, the modulation current is set to 0.2 A. As modulation frequency increases, bandwidth increases first and then decreases, presenting a convex function. Then, as shown in Figure 3b, the TNR increases nonlinearly as modulation frequency increases. The optical spectrum presents an optical frequency comb at a high modulation frequency. However, the changing trend of TNR versus modulation current is opposite to that of TNR versus modulation frequency, and spectral lines appear at a low modulation current. In the time domain, as shown in Figure 3c, the FWHM of optical pulse is not sensitive to modulation frequency. As the addition of modulation current, the FWHM of optical pulse first decreases and then becomes stable.

**Figure 4:**
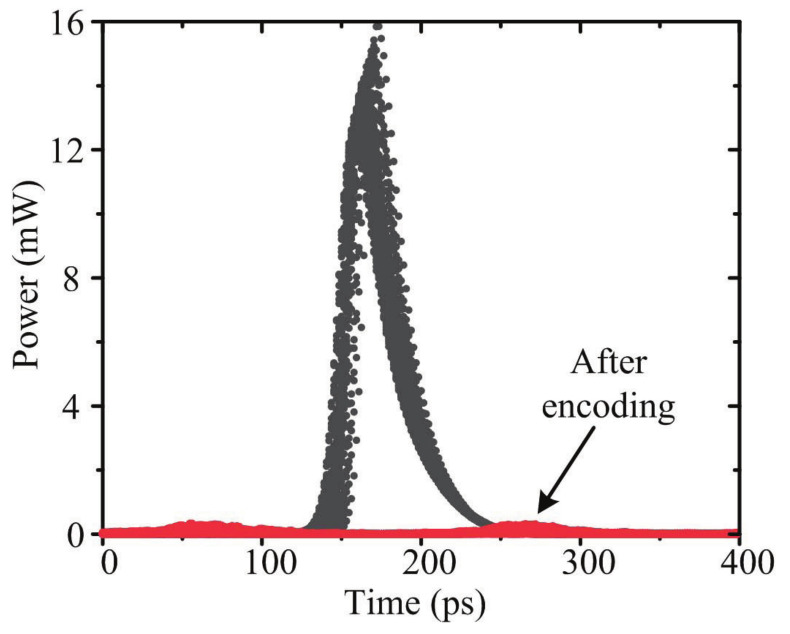
The generated optical stealth carrier before and after encoding.

A simple encoding scheme is adopted for the stealth channel to obtain noiselike characteristics. There are *m* wavelength hopping patterns, and each wavelength pattern has a unique time spread pattern. The time spread pattern is uniformly distributed and the minimum time interval is τ. As a comparison, the generated optical stealth carrier before and after encoding are shown in Figure 4, in which *m* is 8. The bias current is 0.08 A and the modulation current is 0.2 A. Being encoded, the signal is spread over time.

## 3. Optical Stealth Transmission System

**Figure 5:**
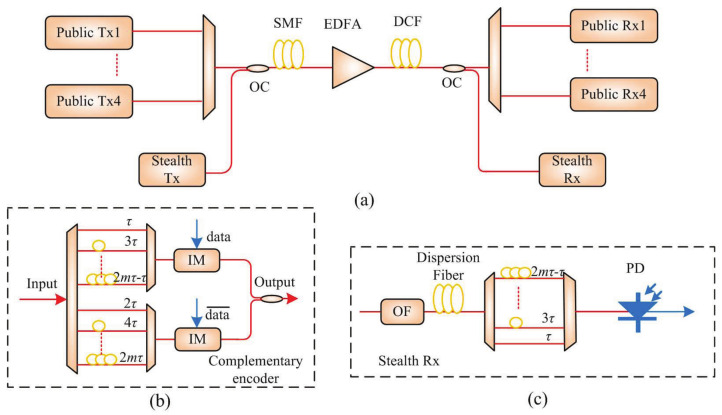
The schematic diagram of optical stealth transmission system based on gain-switched optical pulses. (**a**) The system setup; (**b**) the complementary encoder in stealth transmitter; (**c**) the stealth receiver. Tx, transmitter; OC, optical coupler; SMF, single mode fiber; EDFA, Erbium-doped fiber amplifier; DCF, dispersion compensation fiber; IM, intensity modulator; OF, optical filter; PD, photonic detector.

Based on the optical stealth carrier generator, the setup of optical stealth transmission system is shown in Figure 5a. The public channel has 4 wavelength multiplexed users with a wavelength spacing of 0.16 nm. In the public transmitter, the modulation format of public signal is nonreturn-to-zero (NRZ) and the bit rate is 10 Gbps. The multiplexed signals are transmitted over a 50-km single mode fiber (SMF) span. In the public receiver, the public signals are directly detected after dispersion compensation fiber and de-multiplexer. Through optical couplers, the stealth signal is sent to the public channel and separated from the public channel. Typically, a coded stealth signal is sent during a bit interval to represent 0 bits, and no energy is sent during a bit interval to represent 1 bits[4,9]. Therefore, there is a temporal energy fluctuation when the stealth signal is sent, which exposes the existence of the stealth signal. To avoid the power fluctuation in time domain under continuous 0 bits, a complemented encoding is proposed as shown in Figure 5b. The odd wavelength patterns are modulated by stealth data in the upper branch, while the even wavelength patterns are modulated by inverse stealth data in the lower branch. The insertion loss and time delay of the two branches are equal. In the stealth receiver, as shown in Figure 5c, the public signal and noise are suppressed by an optical filter. Then, the stealth signal is temporally compressed through DCF. The decoder matches the upper branch in the encoder. After decoding, the stealth signal is detected.

## 4. System Simulations and Discussion

### 4.1. Covertness

**Figure 6:**
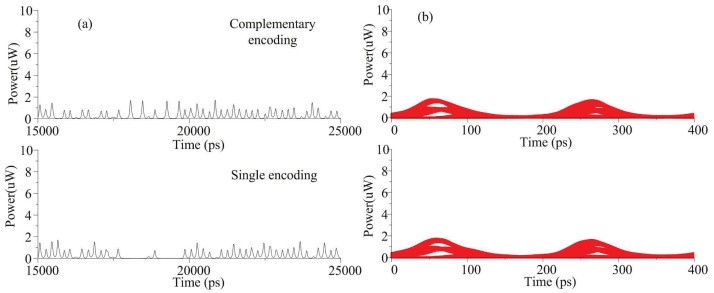
The (**a**) waveform and (**b**) eye diagram of the stealth signal with single and complementary encoding.

During encoding, to avoid temporal fluctuation, a complementary encoding scheme is used in the optical stealth transmission system. The detected waveform comparison between single coding and complementary coding are shown in Figure 6. The bit rate is 2.5 Gbps and the bandwidth of oscilloscope is 10 GHz. The encoded signal with OOK modulation reveals 0 bits due to power discontinuity in time domain. The power of encoded signal with complementary modulation is continuous in time domain. The eye diagram of encoded signal with complementary modulation is the same as the encoded signal with OOK modulation.

**Figure 7:**
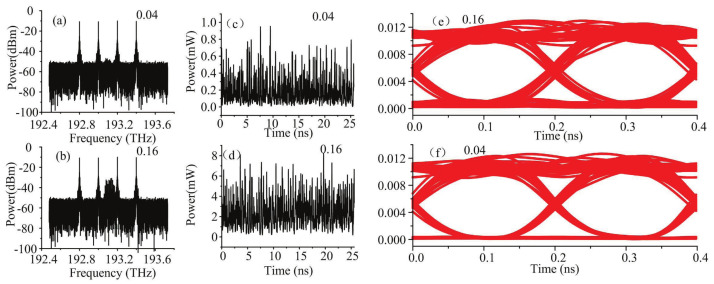
(**a**,**b**) The optical spectrum of public signal, (**c**,**d**) the waveform of stealth channel, and (**e**,**f**) the eye diagram of public channel with different bias currents.

The bias current is the most important factors affecting communication performance of the stealth channel. Therefore, when studying the effect of parameters on covertness, we mainly focus on the bias current. With different bias currents for stealth signal, the optical spectra of public channel are shown in Figure 7a,b. The stealth signal is hidden in the spectral domain when bias current is set to 0.04 A. At 0.16 A, the power of stealth signal power increases, the peak power in the spectral domain is high due to the optical frequency comb characteristics. This high power can easily reveal the presence of the stealth signal. The resolution of the optical spectral analyzer is larger than the symbol rate; the spectral lines cannot be detected in spectral domain. The resolution of the commercial high-precision optical spectral analyzer can reach about 100 MHz. Therefore, for the stealth channel, parameters of the signal generator should be optimized to avoid forming optical frequency combs. Figure 7c,d show the waveform of the stealth signal with different bias currents. After complementary encoding, the stealth signal obtains noiselike temporal characteristics. This feature has not been changed by adjusting bias current. Eye diagrams of the public signal with stealth channel under different currents are shown in Figure 7e,f. When the bias current is 0.16 A, the eye diagram deteriorates due to the increase of signal power. This deterioration can be suppressed by power control in the stealth channel transmitter.

### 4.2. BER Performance

**Figure 8:**
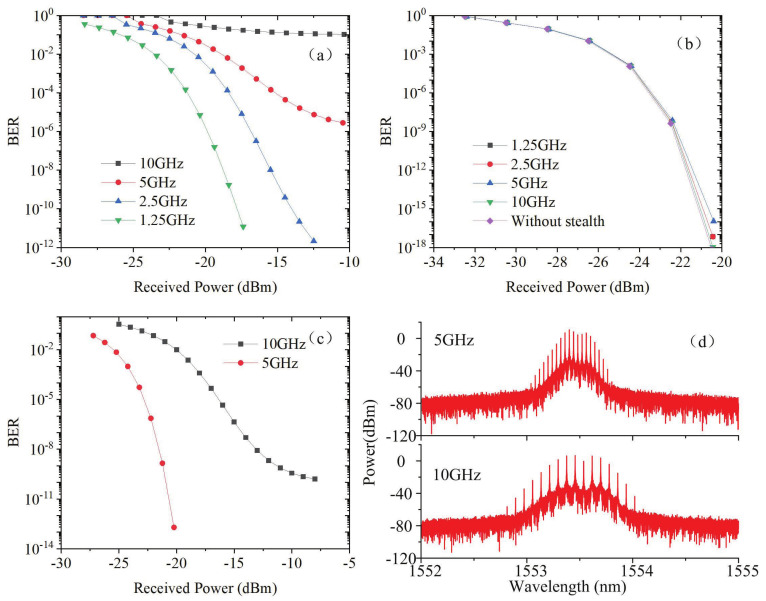
BER performance of (**a**) stealth channel and (**b**) public channels under different frequencies, (**c**) optimum BER results; (**d**) the corresponding optical spectra.

In order to quantitatively analyze the influence of laser parameters on the system performance, the BER performance of the optical stealth transmission system is studied. With different modulation frequencies, the BER curves are shown in Figure 8a. The bias current is 0.08 A and the modulation current is 0.2 A. The BER performance deteriorates with the increase of modulation frequency. The stealth signal can be transmitted error-free over the public channels at 1.25 GHz and 2.5 GHz. Figure 8b shows BER curves of one public channel with stealth signal. The change of the modulation frequency of stealth signal has little effect on the BER performance of public channel. It is verified that the stealth channel has good concealment. After optimizing the laser parameters, the BER results are shown in Figure 8c. Both 10 Gbps and 5 Gbps stealth channel can be transmitted error-free. The bias current is set to 0.13 A and modulation current is set to 0.12 A for the 10 Gbps stealth channel, while the bias current is set to 0.2 A and modulation current is set to 0.1 A for the 5 Gbps stealth channel. However, as shown in Figure 8c, the stealth signal is a clear optical frequency comb in spectral domain. This means the stealth channel has poor concealment in the spectral domain. In addition, it indicates that there is a trade-off between BER performance and covertness of the stealth channel. In order to ensure the spectral covertness of stealth channel, frequency chirp and time jitters of laser outputs are inevitable, and the time jitters will lead to the degradation of BER performance.

**Figure 9:**
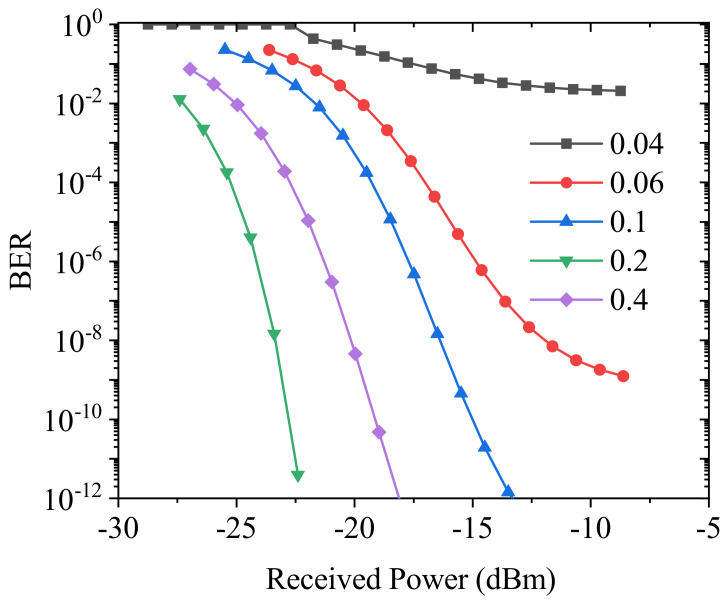
BER of stealth channel with different bias currents.

The BER performance of stealth channel with different bias current is also investigated and the BER curves are shown in Figure 9. As bias current increases, the BER decreases. However, when the bias current increases above 0.2 A, the BER will increase. In addition, the high bias current results in the generation of optical frequency comb and the increase of TNR in the spectral domain. Therefore, the bias current of stealth channel should be optimized in terms of transmission performance and covertness. In terms of covertness, the bias current should be decreased to make the TNR less than 3 dB. In the aspect of transmission performance, channel coding for the stealth channel can be used to reduce the BER; especially, the forward error correction schemes employ iteratively decipherable codes. Due to the superiority of decoder complexity and BER performance, low-density parity-check (LDPC) codes [14,15,16] might be good candidates to significantly improve the BER performance of the stealth channel.

## 5. Conclusions

In this paper, effects of laser parameters on the performance of the optical stealth transmission system based on gain-switched DFB laser are investigated. A complementary encoded optical stealth transmission system is proposed and demonstrated. In the generation of optical stealth carrier, the shape and spectrum of generated optical signal are changed by adjusting laser parameters. Compared with other parameters, bias current has the greatest influence on the temporal characteristics of gain-switched signals. Simulation results show that the power discontinuity in time domain is solved by complementary encoding and the power penalty caused by the stealth signal to the public channel is negligible. In order to enhance the BER performance of the stealth channel, laser parameters can be optimized and adjusted. However, there is a trade-off between BER performance and covertness of the stealth channel. Under the premise of ensuring BER performance of both the stealth and the public channel, parameters should be optimized to reduce the tone-to-noise ratio for the stealth channel. This paper may reinforce the application of the gain-switched laser in photonic layer security of the optical network.

## Figures and Tables

**Table 1:** Some parameter definitions and values in simulations.

Parameter	Value
Confinement factor (Γ)	0.3
Linear material gain coefficient (*a*)	$3.3\times 10^{-20}$ m${}^{2}$
Spontaneous emission rate (β)	$1\times 10^{-4}$
Group velocity ($v_{g}$)	$7.5\times 10^{7}$ ms${}^{-1}$
Electron charge (*e*)	$1.6\times 10^{-19}$ C
Volume of the active region (*V*)	$1.8\times 10^{-16}$ m${}^{3}$
Carrier density at transparency ($N_{t}$)	$1.5\times 10^{24}$ m${}^{-3}$
Nonlinear gain compact factor (ε)	$3\times 10^{-23}$ m${}^{3}$
Photon lifetime ($\tau_{p}$)	$3\times 10^{-12}$ s
Carrier life time at threshold ($\tau_{n}$)	$2.1\times 10^{-9}$ s
Bimolecular recombination coefficient (*B*)	$1.0\times 10^{-16}$ m${}^{3}$ s${}^{-1}$

## Data Availability

The data presented in this study are available on request from the corresponding author.
